# Effects of a Rehabilitation Program Using a Wearable Device on the Upper Limb Function, Performance of Activities of Daily Living, and Rehabilitation Participation in Patients with Acute Stroke

**DOI:** 10.3390/ijerph18115524

**Published:** 2021-05-21

**Authors:** Yun-Sang Park, Chang-Sik An, Chae-Gil Lim

**Affiliations:** 1 Department of Physical Therapy, Seoul Metropolitan Government Seoul National University Boramae Medical Center, Seoul 07061, Korea; yspark@snuh.org; 2 Department of Physical Therapy, Eulji University, Seongnam 13135, Korea; 3 Department of Physical Therapy, Gachon University, Incheon 21936, Korea

**Keywords:** activities of daily living, rehabilitation participation, rehabilitation program, stroke, wearable device

## Abstract

This study investigated the effects of a rehabilitation program using a wearable device on upper limb function, the performance of activities of daily living, and rehabilitation participation in acute phase stroke patients. A total of 44 patients were randomly divided into two groups. The experimental group (n = 22) was requested to wear a glove-type device while they were administered a game-based virtual reality (VR) rehabilitation program of 30 mins per session, 5 sessions per week, for 4 weeks. The program was given in addition to conventional physical therapy. The control group (n = 22) was administered only conventional physical therapy. To examine the intervention effects, the Fugl-Meyer assessment scale, hand strength test, and Jebsen–Taylor hand function tests were performed to examine upper limb function. The Korean version of the modified Barthel Index was used to assess the performance of activities of daily living, and the Pittsburgh rehabilitation participation scale was used to estimate rehabilitation participation. Neither the experimental nor the control group showed significant differences in the pre-intervention homogeneity test, while both groups showed significant improvement in all post-intervention dependent variables. Notably, the experimental group showed a significantly greater improvement in the results of the hand strength test, Jebsen–Taylor hand function test, and Modified Barthel Index. The findings suggest that the rehabilitation program using a wearable device, in addition to conventional physical therapy, is more effective than conventional therapy alone for improving upper limb function, the performance of activities of daily living, and rehabilitation participation in acute phase stroke patients. Our findings suggest that the novel rehabilitation program using a wearable device will serve not only as an effective therapy for enhancing the upper limb function, the performance of activities of daily living, and rehabilitation participation in acute phase stroke patients but also as a highly useful intervention in actual clinical practice alongside conventional physical therapy.

## 1. Introduction

With recent advancements in medical science, increased life expectancy has led to a rise in the size of the older population and in numbers of geriatric diseases and complications. One such geriatric disease is stroke, which is also called a cerebrovascular accident. Stroke is caused by an obstruction in the blood flow to the brain or a hemorrhage. Brain cells deprived of oxygen gradually die, resulting in the loss of abilities regulated by the brain, such as motor control and cognitive function [1]. The consequent functional damages leave various partly permanent disabilities even after recovery, including cognitive, linguistic, sensory, and motor paralysis [2]. In particular, acute phase stroke patients suffer from paretic side hemiakinesia that increases the use of the upper limb on the unaffected side to induce a learned non-use phenomenon in the paretic side upper limb. This reduces the chance of repetitive exercise to facilitate brain reorganization [3], and such upper limb dysfunction makes it difficult for patients to perform basic activities in daily life. It is also a factor leading to permanent damage to the independence of acute phase stroke patients [4].

Therefore, as a therapy for the recovery of paretic side upper limb function in acute phase stroke patients, general muscle strengthening exercise, constraint-induced movement therapy (CIMT), mirror therapy-based active observation, and electrical treatment have been used conventionally [5,6,7]. However, they mostly induce low levels of motivation in patients to initiate voluntary participation in rehabilitation. In CIMT, the psychological pressure from the restricted use of the unaffected side may prompt refusal of treatment, while in mirror therapy-based active observation, the effects may be obtained only when combined with other therapies rather than used alone [6,8].

Barreca et al. [9] and Wolf et al. [10] reported that active exercise, including repetitive and focused training, functional and meaningful task-oriented training, and activities of daily living, exerted positive influences on improving physical function. Virtual reality (VR)-based rehabilitation programs, in particular, have been widely applied in the field of rehabilitation in line with technological advancement. Such programs provide motivation to perform tasks independently by providing training based on sound or voice and virtual environments that closely resemble reality with various visual and auditory feedbacks on-screen while eliciting interest and fun through games suitable to the level of difficulty the patient needs [11,12]. Recent rapid advancement in the VR system strongly emphasizes the accuracy and diversity of sensory feedbacks that would induce an appropriate level of motivation in patients toward active participation and accomplishment and, based on this, enable focused training of tasks required for functional enhancement [13,14,15,16]. In particular, simple manipulation allows various therapeutic programs to provide challenging and specific task-focused training [17].

The RAPAEL Smart Glove is a glove-based software application that can be worn on the hand of a stroke patient who, through each VR-based game, can access a biofeedback system. The wearable device is loaded with 9-axis sensors, including an acceleration sensor, an angular speed channel, and a magnetic sensor, each consisting of three channels. The device can sense forward and backward motions of the forearm, folding/unfolding and left/right displacement of the wrist, and bending and unbending of the fingers, while the five bending sensors can read the degree of finger bending. Data from all sensors are collected and transmitted by the micro-controller. Notably, to improve the learning of various functional tasks, game-based tasks are used to provide the user data of the progression of training, level of interest, and exercise functional scores [18].

Recently, Lee et al. [19] and Buyn et al. [20] reported on the effects of smart gloves on the upper limb and cognitive functions and the performance of activities of daily living in stroke patients. Furthermore, the cognitive reserve may influence the motor outcome by a robotic device intervention [21]. Previous studies focused on the use of a conventional game device, and there is a severe lack of comparative studies that report on the positive effects and effectiveness of a VR rehabilitation program using a wearable device for acute phase stroke patients, with respect to the recovery of upper limb functions, the performance of activities of daily living, as well as the level of rehabilitation participation. In addition, most previous studies investigated VR-based task-focused training in chronic stroke patients, focusing on the increased muscular strength in gross motor areas and leaving factors such as multilateral hand functions, including the fingers below the wrist, unexamined [12,22]. Thus, this study focused on the effects of a rehabilitation program using a glove-type wearable device on the detailed upper limb function, including the wrist and fingers, in acute phase stroke patients, as well as on the performance of activities of daily living and the level of rehabilitation participation.

## 2. Materials and Methods

### 2.1. Participants

The inclusion criteria were as follows: acute phase stroke ≤1 month from the date of onset among hemiplegia patients diagnosed with stroke based on magnetic resonance imaging (MRI) or computed tomography, a score of ≥20 points on the Mini-Mental Status Examination Korean version, and willingness to comply with the therapist’s instructions. We excluded patients who were unable to remain independently seated for ≥30 mins or to manipulate the smart gloves while showing modified Ashworth scale G3 or above for the upper limb; no visual or auditory dysfunction or defect; and no musculoskeletal disorder in the upper limb.

**Figure 1:**
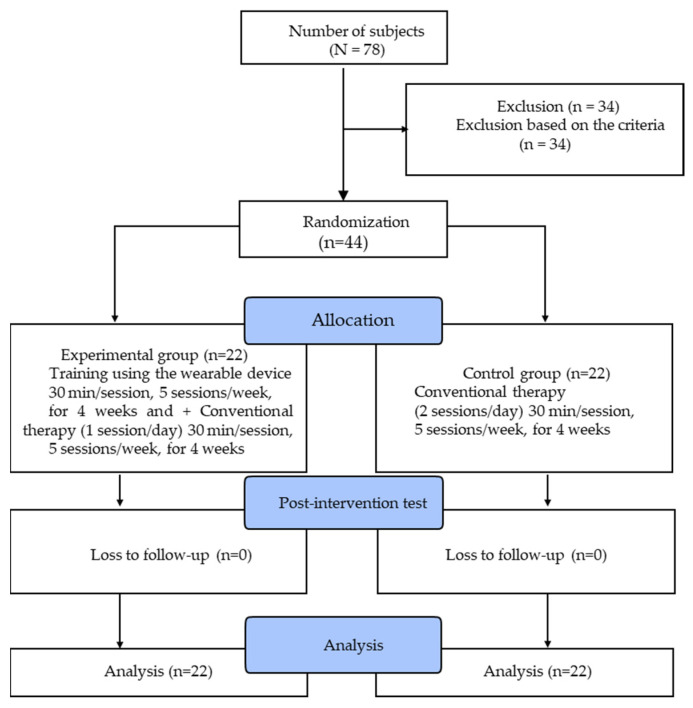
Experimental procedures.

Based on the selection criteria, a total of 44 patients were selected. The general characteristics other than those stated by the criteria are presented in Table 1. The two groups each contained 12 male patients and 10 female patients. In the experimental group, the paretic side was left in 8 patients and right in 14 patients, while those in the control group were left in 9 patients and right in 13 patients. The average age was 60.59 ± 18.12 years in the experimental group and 62.29 ± 13.97 years in the control group. All areas of the homogeneity test on the general characteristics showed no significant difference in the experimental and control groups (*p* > 0.05) (Table 1). The experimental procedures are outlined in Figure 1.

All participants were given a detailed description of the purpose and necessary information regarding the study and were informed that they could withdraw from the study at any time, after which they were requested to sign a consent form. A pre-test was performed 1 day prior to the start of the actual test, and after the end of the 4-week test, a post-test was performed. The study was conducted according to the guidelines of the Declaration of Helsinki and was approved by the Institutional Review Board of Gachon University (1044396-202103-HR-051-01).

### 2.2. Procedures and Intervention

**Figure A1:**
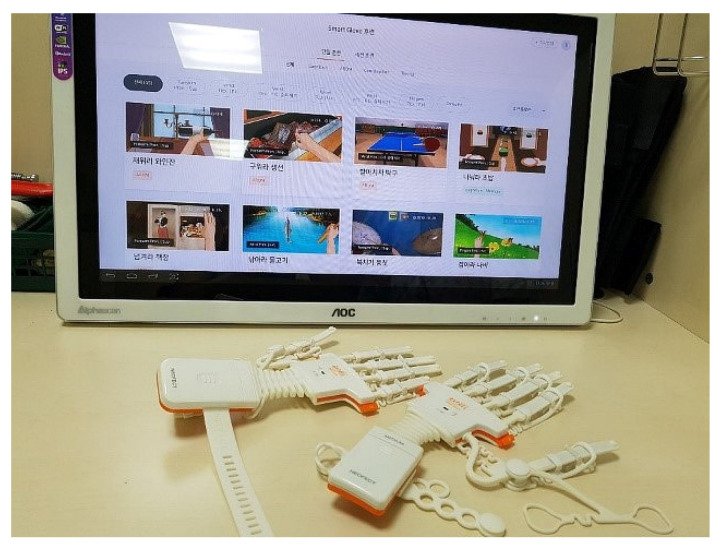
Wearable device (RAPAEL Smart Gloves).

**Figure A2:**
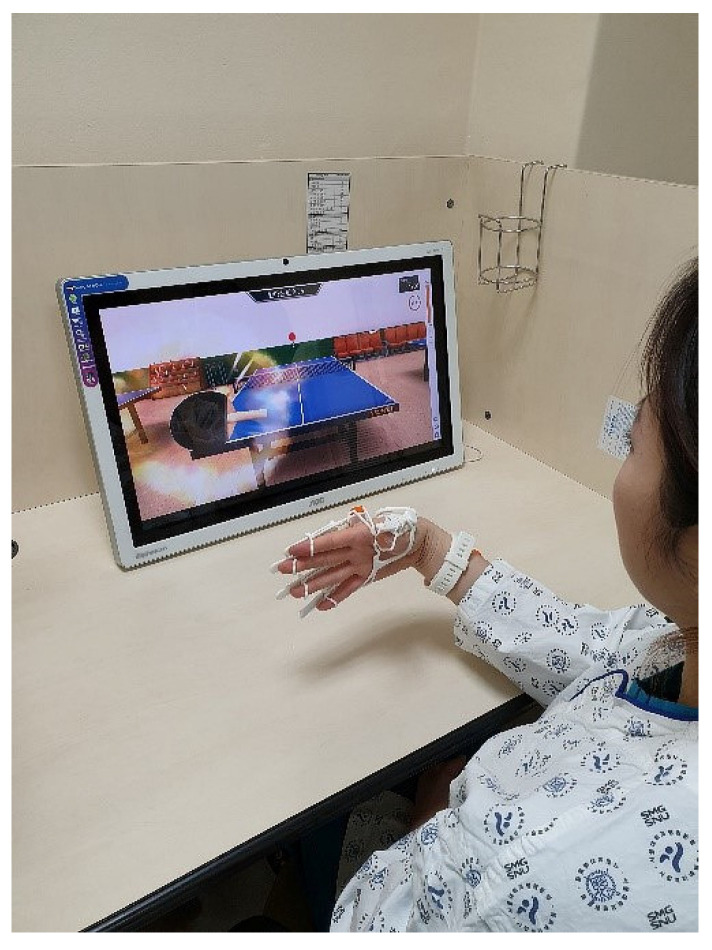
Illustration of the rehabilitation program using a wearable device.

**Figure A3:**
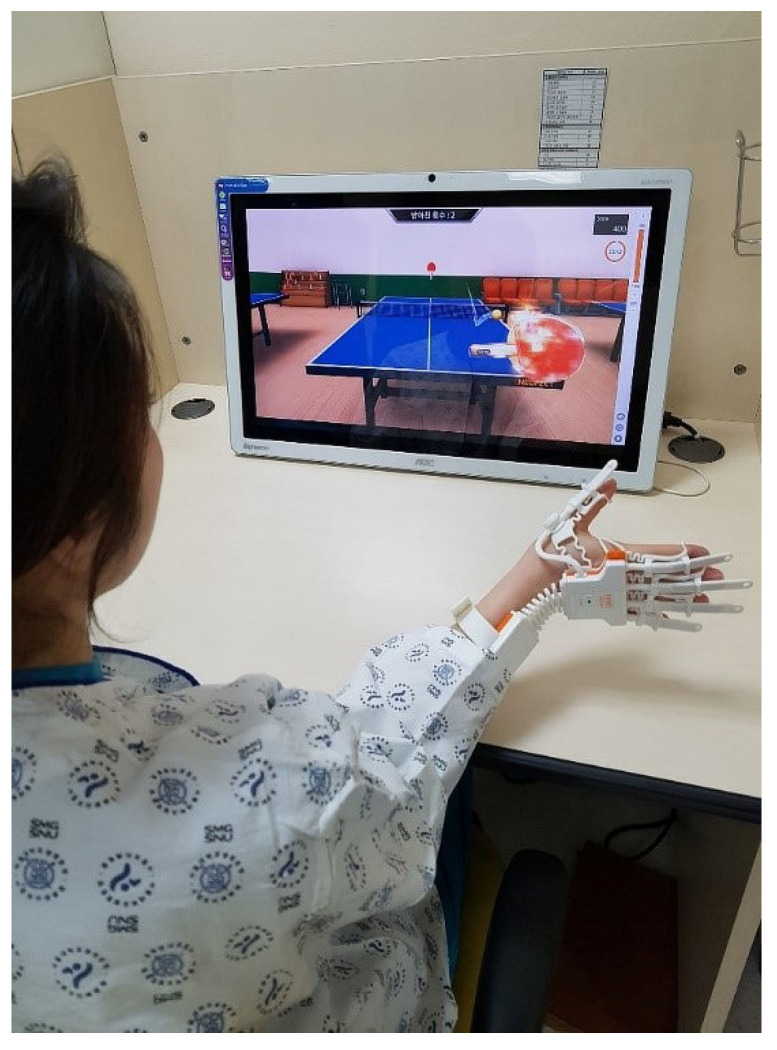
Illustration of the rehabilitation program using a wearable device.

To minimize bias, the 44 subjects were randomly divided into the experimental group (n = 22) and control group (n = 22). The control group was given conventional physical therapy for 30 mins per session, 5 days a week, during the 4-week training period. The conventional physical therapy was based on training to improve upper limb function in stroke patients suggested by Song and Park [23]. According to the patient’s performance ability, training was repeated in consideration of the difficulty level, and assistance was provided. The experimental group was given, in addition to conventional physical therapy, game training of the upper limb training program from RAPAEL Smart GloveTM, for 30 mins per day, 5 days a week, 20 times in total, during the 4-week period. The game-based functional training and activities of daily living (catching butterflies and balls, squeezing an orange, fishing, cooking, floor cleaning, wine pouring, fence painting, and page-turning) were adjusted to the level of difficulty suitable for the patients in the experimental group [24]. The conventional therapy program for the control group was based on the use of exercise and tools related to the passive/active shoulder joint and hand functions for improving upper limb function, while activities of daily living were the basic, instrumental activities in consideration of the joint range of motion and functional abilities (Figure A1, Figure A2 and Figure A3).

### 2.3. Outcome Measurements

Upper Limb Function

For the assessment of upper limb function, the Fugl-Meyer assessment scale (FMA) [25], hand strength test [26], and Jebsen–Taylor hand function test (JTHFT) [27] were performed, while activities of daily living were assessed based on the Korean version of the modified Barthel Index (K-MBI) [28] and the level of rehabilitation participation was estimated based on the Pittsburgh rehabilitation participation scale [29]. The tests were carried out before and after the intervention.

### 2.4. Sample Size Estimation

G power 3.0.1 software (Heinrich Heine University Düsseldorf, Düsseldorf, Germany) was used to determine the sample size. Power calculation was performed using FMA scores from a previous study, which applied VR-based rehabilitation for the upper extremity in stroke survivors, hypothesizing a similar efficacy between our rehabilitation and the previous rehabilitation [30]. A total of 36 participants were estimated to be required with a power for efficacy 80%, and a significance level of 0.05.

### 2.5. Statistical Analysis

All statistical analyses were performed using SPSS ver. 21.0 (IBM Corp., Armonk, NY, USA). For the general characteristics of the study subjects, the mean and standard deviation were calculated via descriptive analysis. For the test of normality, the Shapiro–Wilk test was used. An independent t-test and chi-square test were conducted to compare general characteristics. Repeated Measure ANOVA was conducted to determine whether any interaction existed between groups and time points. For all data, the level of statistical significance was set at α = 0.05.

## 3. Results

Neither the experimental group nor the control group showed significant differences across all pre-intervention homogeneity tests while showing significant improvement in all post-intervention dependent variables. Table 2 summarizes an interaction between group and time effect, the main effect of time, and the main effect of group in outcome variables.

## 4. Discussion

We examined the effects of a game-based rehabilitation program using a wearable device in acute phase stroke patients on hand grip and function, the performance of activities of daily living, and rehabilitation participation. We found significant improvements in the experimental and control groups, while the experimental group exhibited greater improvements than did the control group. Although conventional therapy is a helpful intervention for improving upper limb function and the performance of activities of daily living in acute phase stroke patients, we found that the rehabilitation program using a wearable device could promote functional recovery to a greater degree. Notably, the results indicated an improvement in treatment satisfaction, which may be attributed to the following factors: the performance has to be accurate to a set timing during the training period, and even for incorrect performance, visual and auditory feedback are immediately provided to ensure self-learning that demands various hand movements, while participation in exercise is improved and task performance can be directly observed by the patient.

Some stroke patients experience difficulties in performing activities of daily living independently due to upper limb dysfunction [31]. The rehabilitation of such stroke patients requires appropriate motivation toward active participation and accomplishment based on interest and attention, as well as focused training on the tasks essential in functional improvement. On this premise, with the recent revolutionary technological advancement that led to the focus on VR technology in diverse fields, studies have proactively investigated the use of VR in the field of rehabilitation for stroke patients. The rehabilitation program applying a VR-based game, in particular, has the benefit of motivating patients to participate in more functional activities while increasing the interest and immersion in rehabilitation, and several other effects such as stress relief are expected [32,33]. The program also increases the self-complacency in patients by motivating them toward task accomplishment based on the interactions [34].

Kim [11] facilitated upper limb functional recovery and brain reorganization as assessed by the test of upper limb function and functional MRI, after performing an upper limb exercise program using a VR game for chronic stroke patients. However, their study differed from the present study in that the former provided the game program as a tool created purely for the purpose of rehabilitation training rather than to induce interest in study subjects.

Lee et al. [35] used a balance exercise based on a VR program called BioRescue and reported an improvement in the performance of activities of daily living, which agreed with the results in this study. However, their program focused on exercise time and intensity control rather than providing a game to raise interest, so that the observed improvement in daily activities was the result of exercise rather than increased interest and immersion.

Compared with other previous studies mainly using the common VR game devices such as Nintendo Wii, Play-Station, and XBOX [36,37,38], the device used in the present study was a light-weight and precise wearable device that can be directly put on the paretic hand of the patient while providing visual and auditory feedback and inducing movements required for performing daily activities, which distinguished it from the devices used in previous studies as it enabled the subjects to use their hands in far more diverse ways.

Yin et al. used VR-based training in 23 patients within 1 month of the onset of stroke, and its effects were compared with the conventional intervention [39]. The post-intervention FMA scores were shown to have increased in all participants, while no change was observed in other assessment tools. This led them to conclude that the applied training was not more outstanding than the conventional intervention, as opposed to the findings of the present study. However, they applied a short intervention period (2 weeks), which was thought to have led to a contrasting conclusion.

As can be seen, previous studies regarding the effects of a VR rehabilitation program on the upper limb function in stroke patients report either significant improvement [40], or no improvement [41,42], which prevents the drawing of any definite conclusion; thus, further studies are warranted in line with future technological advancement. In the most recently reported meta-analysis, Rutkowski et al. [13] reported that the use of specialized virtual reality and gaming virtual reality could be advantageous for treatment of the upper extremity, but not for hand dexterity and gait in all pathologies considered. Specialized virtual reality can improve balance in neurological patients. Karamians et al. [14] reported that the VR- or gaming-based upper extremity rehabilitation poststroke appears to be more effective than conventional methods. Further in-depth study of the variables affecting improvement, such as individual motor presentation, treatment dose, and the relationship between them, is needed. In addition, Domínguez-Téllez et al. [15] reported the potential benefits of VR interventions on the recovery of UL motor function (FMA) and on quality of life after stroke. Furthermore, robotic treatment using a set of four devices significantly improved UL motor function, activities, and participation in subjects with subacute stroke to the same extent as a similar amount of conventional therapy [43].

The findings in this study collectively suggested that a rehabilitation program using a wearable device is effective for enhancing the upper limb function in acute phase stroke patients as well as their performance of activities of daily living and rehabilitation participation. Nonetheless, the limited space and number of subjects mean that the results cannot be generalized yet to all acute phase stroke patients. Moreover, whether the effects of natural recovery after the onset of stroke had any influence on the observed results remains unknown. For this reason, follow-up monitoring to verify the consistency of intervention effects could not be carried out. Furthermore, the program setting should be more specific in adjusting the level of difficulty to ensure more efficient intervention for the treatment process. Finally, the detailed subjective quality of life in acute phase stroke patients based on the enhanced upper limb function and rehabilitation participation could not be estimated. Thus, further studies should recruit patients with more diverse clinical features, and a follow-up study should focus on specific factors that could enhance the quality of life.

## 5. Conclusions

We investigated the effects of a rehabilitation program using a wearable device on upper limb function, activities of daily living, and rehabilitation participation in acute phase stroke patients. We found that, compared with the group that performed only conventional physical therapy, the group that performed the rehabilitation program using a wearable device in addition to conventional physical therapy showed significantly higher improvement with respect to hand function, the performance of activities of daily living, and rehabilitation participation. Notably, compared with the wearable device used in previous studies, the wearable device in the present study involves patients directly, i.e., patients wear the gloves to perform diverse hand movements while ensuring a sensory experience that resembles a direct experience for enhanced immersion and enjoyable participation. The program is anticipated to be an intervention with positive clinical effects for enhancing the upper limb function in acute phase stroke patients as in the neurodevelopmental therapy, CIMT, and mirror therapy-based active observation.

## Figures and Tables

**Table 1:** General characteristics of study subjects and result of the homogeneity test (N = 44).

Category	Experimental Group (n = 22)	Control Group (n = 22)	χ^2^/*t* (p)
Sex, male/female (%)	12/10 (54.5/45.5) ^a^	12/10 (54.5/45.5)	−0.298 (0.767)
Paretic side, right/left (%)	14/8 (68.2/31.8)	13/9 (54.5/45.5)	−0.303 (0.764)
Age (years)	60.59 ± 18.12	62.29 ± 13.97	−1.009 (0.319)
Height (cm)	160.31 ± 10.55	161.53 ± 8.57	−0.420 (0.676)
Weight (kg)	59.52 ± 11.47	57.40 ± 11.37	0.615 (0.542)
Length of stay (days)	17.73 ± 5.98	16.82 ± 7.28	0.453 (0.653)
MMSE-K (point)	21.91 ± 4.80	22.45 ± 4.86	−0.375 (0.710)

^a^ Number of subjects (composition), values are expressed as mean ± standard deviation.

**Table 2:** Baseline and post-intervention in upper limb function, the performance of activities of daily living, and rehabilitation participation before and after training (N = 44).

Variables		Experimental Group (n = 22)		Control Group (n = 22)		Time × Group		
		Pre	Post	Pre	Post	F	*p*	η_p_^2^
Upper limb function								
Fugl-Meyer assessment scale		66.50 ± 24.43	87.95 ± 14.16	62.95 ± 28.81	86.00 ± 15.97	0.123	0.728	0.003
Hand strength test	Grip power	18.68 ± 15.85	31.50 ± 18.46	16.50 ± 21.51	24.88 ± 26.39	4.135	0.048	0.090
	Palmar pinch	2.86 ± 3.36	8.00 ± 5.17	3.38 ± 4.09	6.40 ± 5.46	4.346	0.043	0.094
	Lateral pinch	5.18 ± 4.23	9.90 ± 5.99	5.00 ± 5.16	7.02 ± 6.72	5.831	0.020	0.122
	Tip pinch	2.41 ± 3.02	5.45 ± 4.29	2.63 ± 2.92	4.18 ± 3.98	5.595	0.023	0.118
Jebsen–Taylor hand function test		14.09 ± 15.63	39.91 ± 29.55	20.68 ± 22.74	33.04 ± 27.06	6.893	0.012	0.141
Activities of daily living								
Korean version of the modified Barthel Index		46.00 ± 25.83	77.68 ± 19.79	49.55 ± 19.88	71.18 ± 17.94	4.318	0.044	0.093
Rehabilitation participation								
Pittsburgh rehabilitation participation scale		3.50 ± 1.10	3.95 ± 1.13	3.82 ± 1.00	4.23 ± 0.81	0.042	0.839	0.001

Values are expressed as mean ± standard deviation. Notably, there were significant differences in hand strength test, JTHFT, and K-MBI between the two groups over time. However, there were no significant interactions between time and group in FMA and Pittsburgh rehabilitation participation scale. Significant main effect of time was found in both variables (*p* < 0.001, η_p_^2^ = 0.696; *p* < 0.001, η_p_^2^ = 0.265, respectively) but no significant group effect was found (*p* = 0.656, *p* = 0.309, respectively).

## Data Availability

This study is available on request from the corresponding author. The data are not publicly available due to privacy or ethical restrictions.
